# Activities contributing to energy expenditure among Guatemalan adults

**DOI:** 10.1186/1479-5868-4-48

**Published:** 2007-10-02

**Authors:** Cria O Gregory, Manuel Ramirez-Zea, Reynaldo Martorell, Aryeh D Stein

**Affiliations:** 1 Nutrition and Health Sciences Program, Graduate Division of Biological and Biomedical Sciences, Emory University, Atlanta, GA, USA; 2 Institute of Nutrition of Central America and Panama (INCAP), Guatemala City, Guatemala; 3 Hubert Department of Global Health, Rollins School of Public Health, Emory University, Atlanta, GA, USA

## Abstract

**Background:**

Guatemala has experienced a substantial increase in overweight and obesity in recent years, yet physical activity patterns and consequent energy expenditure are largely unexplored in this population.

**Methods:**

To describe overall physical activity levels (PAL) and activities contributing to daily energy expenditure, we analyzed time spent in daily activities as reported by 985 women and 819 men, living in rural and urban areas of Guatemala in 2002–04.

**Results:**

Physical activity levels recommended to prevent obesity (PAL ≥ 1.70) differed by residence/occupation among men (agricultural-rural: 77%; nonagricultural-rural: 36%; urban: 24%; P < 0.01), but not women (rural: 2%; urban: 3%; P = 0.5). Median energy expenditure was higher among agricultural-rural men (44 MET*h/d; MET = metabolic equivalent) compared to nonagricultural-rural (37 MET*h/d) and urban men (35 MET*h/d; P < 0.01); energy expenditure was slightly lower among rural compared to urban women (34 MET*h/d vs. 35 MET*h/d; P < 0.01). Occupation was the largest contributor to energy expenditure (19–24 MET*h/d); among women and nonagricultural-rural and urban men this was primarily of a light intensity. Energy expenditure in sedentary activities ranged from 2 MET*h/d among rural women to 6 MET*h/d among agricultural-rural men. Any sports/exercise time was reported by 35% and 5% of men and women, respectively. Nevertheless, the majority of participants believed they were significantly active to stay healthy.

**Conclusion:**

Overall, energy expenditure was low in the population not dedicated to agricultural occupations; an increased focus on active leisure-time behaviors may be needed to counterbalance reductions in energy expenditure consequent to sedentarization of primary occupations.

## Background

No longer limited to developed countries, overweight and obesity have become a global epidemic [1]. This excess in adiposity develops from a chronic positive imbalance between energy intake and energy expenditure [2]. In developing countries, shifts away from traditional diets and increased consumption of sugar, fat, refined foods, and total energy have been well documented [3,4]; less is known about trends in energy expenditure. In addition to its role in preventing obesity, a physically active lifestyle can aid in the prevention of cardiovascular disease, type 2 diabetes, osteoporosis, some cancers, and depression [5-7]. An estimated 119,000 deaths in Latin America in 2000 were attributed to an inactive lifestyle [8].

Four primary domains of daily activity provide opportunities for energy expenditure: occupation, transportation, domestic chores, and leisure-time [8], while physical inactivity is characterized by sedentary activities that require minimal energy expenditure [1]. It is generally believed that activities of daily living in developing countries demand greater energy expenditure than those in more developed countries [1,4]; these include walking or cycling for transportation, carrying water, farming and tending livestock, and collecting and preparing fuel for cooking and heating. However, recent trends in developing countries towards industrialization and modernization, particularly in urban areas, have created environments that favor less-demanding physical activities [9,10]. Physical activity research in Latin America has tended to focus on leisure-time [11-13] or other specific activities [14], yet several studies have indicated that failing to account for transportation, occupation, and domestic activities can seriously bias estimates of physical activity in developing countries [15,16]. Additionally, over the past several decades the health benefits of consistent moderate-intensity activities, which may come from any of the four domains of activity, have become well accepted [17].

Given the multitude of health benefits related to physical activity and the recent changes in the environment and social structure of developing countries experiencing epidemiologic and nutritional transitions, it is essential to assess the contributions of various domains of activity to total energy expenditure, and to investigate differences by residence, occupation, and sex in levels and components of physical activity. Thus, our objectives were 1) to describe the physical activity patterns of a sample of women and men living in rural and urban areas of Guatemala, and 2) to describe the contribution of different activity domains to their daily energy expenditure.

## Methods

### Population

We have prospectively followed a cohort of Guatemalan women and men who participated in a nutritional supplementation trial conducted by the Institute of Nutrition of Central America and Panama (INCAP) in four rural villages in Guatemala from 1969–1977 [18]. Several rounds of follow-up have been conducted. In the 2002–04 follow-up, the target sample included all individuals who had participated as children in the original study (n = 2392), and their spouses. Of the 2392 who participated as children, 274 had died, 162 were living out of the country, and 101 were untraceable. Of the remaining 1855, 1493 (80% participation) completed a physical activity questionnaire; of those, 1308 also completed the anthropometric assessment (71% participation). Additionally, we obtained physical activity and anthropometric data from 576 spouses. We excluded from the present analysis participants who were missing covariate data or women that were pregnant, for a final sample of 1804 (819 men; 985 women). Excluded participants were slightly younger (31.2 vs. 32.7 y, P < 0.01) and more likely to be living in rural areas (82% vs. 70%, P < 0.01). All data collection was approved by review boards at both INCAP and Emory University.

### Physical activity assessment

Field workers administered a physical activity questionnaire asking about the frequency and duration of activities performed over the preceding year on a typical workday; 92% of women and 71% of men reported participating in their primary occupation 6–7 days/week. Activities included time spent sleeping, on personal needs, on household chores, in transportation to work (walking, biking, or riding in a car or bus), at primary and secondary occupations, walking carrying laundry, firewood, or water, walking to church or for errands, playing sports or exercising, and in sedentary activities (watching television, in a hammock, reading, studying, etc). We multiplied the duration of each reported activity by its intensity, or metabolic equivalent (MET), which is a multiple of the basal metabolic rate [19,20]. One MET is approximately equal to 1 kcal/kg body weight/h for a person weighing 60–70 kg. If cumulative time in all reported activities was less than 24 h we assigned a MET of 1.4 (light/sedentary activities), equivalent to light activity while sitting, for the residual time [20,21]; if more than 24 h, then time in each activity was prorated to its proportion of total reported time. The range of reported time was 942.0 – 2039.9 min, with a mean and standard deviation of 1417.1 ± 87.6 min. We calculated individual physical activity level (PAL) by averaging MET*h over 24 h. A 24 h PAL ≥ 1.70 throughout life is recommended to avoid obesity [20]. We classified chores with MET ≥ 3.0 as moderate/vigorous, and with MET < 3.0 as light intensity [22]. The methodology and MET values for quantifying occupational activity were based on the FAO/WHO/UNU report of Energy and Protein Requirements [20]; as suggested in this report, we assumed that for moderate occupations 25% of the time would be spent sitting or standing and 75% performing the specific occupation, while for vigorous occupations the distribution would be 40% and 60%, respectively. Following these calculations we classified occupations with MET ≥ 2.3 as moderate/vigorous, and with MET < 2.3 as light intensity [20]. We categorized transportation as walking/cycling versus riding in a car/bus; all reported sports were of a vigorous intensity.

Questionnaires administered 12 weeks apart by two different fieldworkers among 17 men and 16 women (all rural) showed high reproducibility among both men (r = 0.9, p < 0.01) and women (r = 0.8, p < 0.01). Comparison of the questionnaire against two nonconsecutive days of heart rate monitoring (22 men; 19 women) was good for men (r = 0.6, p < 0.01), but less so for women (r = 0.04, ns) [23].

### Analysis of energy expenditure

We grouped activities into the primary domains of daily activity (sleep, occupation, chores, transportation, sport/exercise, and sedentary activities), and further stratified these by intensity level in order to quantify MET*h/d expended in various activities.

### Analysis of time in activities

We summed time in domains of activity and divided by total time, in order to calculate the proportion of time spent in various activities. We further grouped moderate/vigorous activities across all domains to calculate total min/d, and calculated the proportion of individuals who spent ≥ 60 min/d in moderate/vigorous activity [24].

### Anthropometric assessment

Weight and height measures were obtained in duplicate by trained field workers. Where discrepancies occurred, a third measure was taken and the closest two measures were averaged. We calculated BMI (kilograms/meter^2^), and categorized BMI ≥ 25 kg/m^2 ^as overweight, and BMI ≥ 30 kg/m^2 ^as obese [25].

### Residence/occupation

We categorized participants residing in Guatemala City as urban, and those residing in one of the original four study villages as rural. Participants living elsewhere in Guatemala were categorized as rural or urban based on answers to a questionnaire of household and neighborhood amenities. We further stratified rural men by primary occupation, and classified them as agricultural or nonagricultural (any occupation other than farming) in order to distinguish men maintaining more traditional labor activities. Eighty-one percent of women reported housewife as their primary occupation, with fewer than 1% reporting agricultural activities. Thus there was insufficient variation to stratify women by occupation.

## Results

At the time of interview most participants were 24–49 years old and living in rural areas (Table 1). Approximately 42% of men and 56% of women had not completed primary school (6 years). Overweight (BMI ≥ 25 kg/m^2^) was common among both men and women (44% and 62%, respectively), as was obesity (BMI ≥ 30 kg/m^2^) among women (24%). Most women had given birth multiple times.

**Table 1:** Sample characteristics, by sex^1^

	**Men n = 819**	**Women n = 985**
Age (y)	33.9 ± 6.3	31.7 ± 5.3
Urban (%)	28.5	30.6
Education (%)		
< 6 years	42.4	55.5
6–9 years	44.3	34.4
> 9 years	13.3	10.1
BMI (kg/m2) (%)		
≥ 25	44.3	61.7
≥ 30	10.5	23.6
Number of births (%)		
0	n/a	8.2
1–3	n/a	56.1
>3	n/a	35.6

^1^Mean ± SD or prevalence (%)

Among both men and women, urban residents were more likely to be overweight than other residence-occupation groups (Table 2). Physical activity levels were the highest among agricultural-rural men, followed by nonagricultural-rural men. Approximately three-quarters of agricultural-rural, one-third of nonagricultural-rural, and one-quarter of urban men had PAL ≥ 1.70. Nearly all women were sedentary, with less than 3% of both rural and urban women having PAL ≥ 1.70. Among men, agricultural-rural had the highest, and urban the lowest total energy expenditure; energy expenditure was slightly lower among rural compared to urban women. This same pattern was apparent in time spent in moderate/vigorous activities. Nearly all agricultural-rural men spent ≥ 60 min/d in moderate/vigorous activities, compared to 63% of nonagricultural-rural men, 39% of urban men, 12% of rural women and 22% of urban women. About one-third of men participated in some form of sports or exercise; while overall participation was much lower among women, it was more common among urban than rural women (9% vs. 4%, respectively). Most respondents reported belief that their own level of activity was adequate to maintain health: reported by 86%, 73%, and 57% of agricultural-rural, nonagricultural-rural, and urban men, and 81% and 53% of rural and urban women, respectively.

**Table 2:** Anthropometry and physical activity characteristics, by sex and residence-occupation^1^

	**MEN (n = 819)**				**WOMEN (n = 985)**		
	**Agricultural rural**	**Nonagricultural rural**	**Urban**	**P**^2^	**Rural**	**Urban**	**P**^2^

	**n = 205**	**n = 381**	**n = 233**		**n = 684**	**n = 301**	
Overweight (≥ 25 kg/m^2^) (%)	26.8	44.9	58.8	<0.01	59.8	70.6	0.05
Obese (≥ 30 kg/m^2^) (%)	6.3	10.0	15.0	0.01	24.4	21.3	0.4
24 hr physical activity level (PAL)	1.83 (0.3)	1.56 (0.5)	1.48 (0.3)	<0.01	1.42 (0.07)	1.43 (0.07)	0.02
PAL ≥ 1.70 (%)	77.1	36.0	24.0	<0.01	2.3	3.0	0.5
Energy expenditure (kcal/d)	2638.3 (356)	2241.2 (674)	2127 (416)	<0.01	2041.5 (97)	2059.0 (106)	0.02
Min/d in moderate/vigorous activity	580.6 (215)	95.3 (501)	35.9 (231)	<0.01	10.0 (17.4)	16.8 (41)	<0.01
≥ 60 min/d moderate/vigorous (%)	99.5	62.7	38.6	<0.01	11.7	22.3	<0.01
Any sport/exercise participation (%)	31.7	39.4	29.2	0.2	3.5	9.1	<0.01
Believe sufficient activity (%)							
Yes	86.3	73.0	57.1		81.1	52.5	
No/Don't know	13.7	27.0	42.9	<0.01	18.9	47.5	<0.01

^1^Values are median (interquartile range) or percentage, as appropriate

^2^Kruskal-Wallis test for continuous variables and chi-square test for dichotomous variables

**Figure 1:**
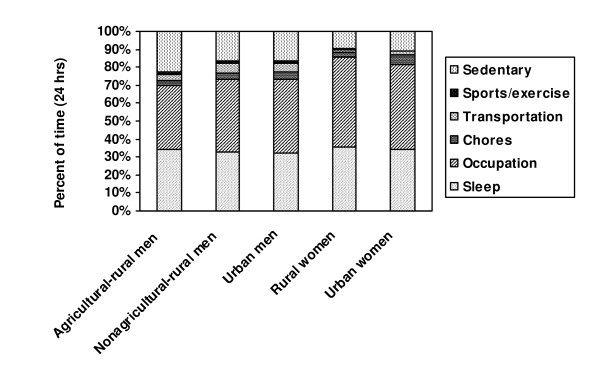
**Mean distribution of time (24 h) in domains of activity, by sex and residence-occupation**. Percent of time per day spent in 6 domains of activity (sleep, occupation, chores, transportations, sports/exercise, and sedentary activities), stratified by sex and residence-occupation.

Time spent sleeping accounted for 32% (urban men) to 35% (rural women) of the 24 h period (approximately 7.5 – 8.5 h; Figure 1). Agricultural-rural men spent the smallest proportion of the day in occupational activities (35%), compared to 40% and 41% among nonagricultural-rural and urban men, and 50% and 47% among rural and urban women, respectively. Chores accounted for 3% of time in daily activities among agricultural-rural men and rural women, 4% among nonagricultural-rural and urban men, and 6% among urban women. Transportation accounted for 4%, 6%, and 5% of time among agricultural-rural, nonagricultural-rural, and urban men, and for 1% and 2% among rural and urban women, respectively. Sports/exercise accounted for 2% of time among agricultural-rural men and 1% among both nonagricultural-rural and urban men; time in sports/exercise was negligible among women. Agricultural-rural men spent the most time in sedentary activities (23%), followed by nonagricultural-rural and urban men (both17%); rural and urban women spent 10% and 11% of the day in sedentary activities, respectively.

Estimates of energy expenditure (MET*h/d) by domains of activity and intensity level are presented in Tables 3 (men) and 4 (women). Agricultural-rural men had the highest and urban men the lowest levels of energy expenditure in occupation, transportation, and sports/exercise; energy expended in sedentary activities was also highest among agricultural-rural men, and similar among nonagricultural-rural and urban men. Energy expended in chores was highest among nonagricultural-rural men. Examining domains of activity by intensity level, agricultural rural men expended the most energy in moderate/vigorous occupations, while urban men expended the most energy in low intensity occupations. Non-agricultural rural and urban men expended more energy in low intensity chores and riding in a car or bus, while agricultural-rural men expended more energy walking or cycling for transportation.

**Table 3:** Energy expended (MET*h/d) in activities of varying intensity among Guatemalan men, by residence-occupation^1^

	**MEN (n = 819)**									
	
	**Agricultural-rural (n = 205)**			**Nonagricultural-rural (n = 381)**			**Urban (n = 233)**			
	**Mean**	**Median**	**25**^th^**, 75**^th^	**Mean**	**Median**	**25**^th^**, 75**^th^	**Mean**	**Median**	**25**^th^**, 75**^th^	**P**^2^
Sleep	8.3	8.0	7.5, 9.0	7.8	8.0	7.0, 9.0	7.7	8.0	7.0, 8.5	<0.01
Occupation	22.5	24.3	17.6, 26.6	20.6	19.9	15.3, 24.1	19.8	18.6	15.3, 24.1	<0.01
*Light*	*0.7*	*0.0*	*0.0, 0.0*	*11.2*	*13.3*	*0.0, 18.3*	*13.5*	*15.6*	*8.1, 21.6*	*<0.01*
*Moderate/vigorous*	*21.8*	*24.3*	*15.8, 26.6*	*9.3*	*0.0*	*0.0, 24.1*	*6.3*	*0.0*	*0.0, 6.5*	*<0.01*
Chores	1.5	0.7	0.5, 1.4	1.9	1.0	0.6, 1.7	1.7	1.0	0.6, 1.6	<0.01
*Light*	*0.7*	*0.6*	*0.4, 0.8*	*1.1*	*0.9*	*0.6, 1.3*	*1.2*	*1.0*	*0.6, 1.3*	*<0.01*
*Moderate/vigorous*	*0.8*	*0.0*	*0.0, 0.0*	*0.8*	*0.0*	*0.0, 0.0*	*0.6*	*0.0*	*0.0, 0.0*	*0.3*
Transportation	3.2	2.5	1.5, 4.4	2.7	2.3	1.1, 3.9	2.4	2.0	1.1, 3.2	<0.01
*Riding car or bus*	*0.1*	*0.0*	*0.0, 0.0*	*1.2*	*0.6*	*0.0, 1.8*	*1.0*	*0.6*	*0.0, 1.7*	*<0.01*
*Walking or cycling*	*3.1*	*2.5*	*1.2, 4.3*	*1.6*	*1.1*	*0.5, 2.0*	*1.4*	*0.9*	*0.3, 2.0*	*<0.01*
Sports/exercise^3^	1.8	0.0	0.0, 2.7	1.3	0.0	0.0, 0.0	0.9	0.0	0.0, 1.2	<0.01
Sedentary activities	6.6	6.2	4.6, 8.2	5.0	4.8	3.3, 6.4	5.0	4.8	3.3, 6.4	<0.01
Total	44.0	44.0	41.1, 47.0	39.2	37.4	34.0, 45.2	37.6	35.4	33.2, 40.1	<0.01

^1^Values are mean, median, and 25^th ^and 75^th ^percentiles of MET*h/d expended in each activity

^2^Kruskal-Wallis test

^3^All sports or exercise reported by participants were of a vigorous intensity

**Table 4:** Energy expended (MET*h/d) in activities of varying intensity among Guatemalan women, by residence^1^

	**WOMEN (n = 985)**						
	
	**Rural (n = 684)**			**Urban (n = 301)**			
	**Mean**	**Median**	**25**^th^**, 75**^th^	**Mean**	**Median**	**25**^th^**, 75**^th^	**P**^2^
Sleep	8.5	8.0	8.0, 9.0	8.3	8.0	7.5, 9.0	<0.01
Occupation	20.5	20.8	18.8, 22.6	19.2	19.7	17.0, 22.0	<0.01
*Light*	*20.2*	*20.6*	*19.7, 22.4*	*19.1*	*19.6*	*17.0, 21.9*	*<0.01*
*Moderate/vigorous*	*0.3*	*0.0*	*0.0, 0.0*	*0.1*	*0.0*	*0.0, 0.0*	*0.5*
Chores	1.5	0.8	0.5, 1.2	2.8	1.2	0.8, 2.6	<0.01
*Light*	*0.9*	*0.8*	*0.5, 1.1*	*1.2*	*1.1*	*0.7, 1.5*	*<0.01*
*Moderate/vigorous*	*0.5*	*0.0*	*0.0, 0.0*	*1.5*	*0.0*	*0.0, 0.3*	*<0.01*
Transportation	0.9	0.5	0.2, 1.1	1.1	0.7	0.3, 1.3	<0.01
*Riding car or bus*	*0.1*	*0.0*	*0.0, 0.0*	*0.3*	*0.0*	*0.0, 0.0*	*<0.01*
*Walking or cycling*	*0.8*	*0.5*	*0.2, 1.0*	*0.8*	*0.6*	*0.3, 1.1*	*0.1*
Sports/exercise^3^	0.1	0.0	0.0, 0.0	0.2	0.0	0.0, 0.0	0.02
Sedentary activities	2.9	2.4	1.7, 3.6	3.3	3.0	2.0, 4.3	0.03
Total	34.4	34.0	33.2, 34.9	34.7	34.5	33.5, 35.2	<0.01

^1^Values are mean, median, and 25^th ^and 75^th ^percentiles of MET*h/d expended in each activity

^2^Kruskal-Wallis test

^3^All sports or exercise reported by participants were of a vigorous intensity

Rural women had higher energy expenditure levels in sleep and occupational activities, while urban women had higher energy expenditure levels in chores, transportation, sports/exercise, sedentary activities, and overall. Almost no energy expended in occupations was at a moderate/vigorous intensity among either rural or urban women. Urban women expended more energy riding in a car or bus and in both low and moderate/vigorous intensity chores.

The 3 most common occupations among nonagricultural-rural men were construction worker (25%), driver (8%), and police officer/security guard (7%), and among urban men were construction worker (13%), salesman (7%), and industrial machine operator (6%; data not shown). The overwhelming majority of women (85% rural and 71% urban) reported housewife as their primary occupation. Among participants who did report sports/exercise, the most common activities among men were soccer (78%), running (6%), basketball (4%), and lifting weights (3%); and among women were basketball (22%), aerobics (18%), running (18%), gymnastics (14%), and soccer (12%).

## Discussion

Overweight and obesity has become common in this population [26], particularly among women. The situation is likely to become worse, as only 2% of women and 43% of men were meeting activity levels recommended for preventing obesity. Despite spending more time in sedentary activities than any other resident-occupation group, the physically demanding nature of their occupational activities allowed for agricultural-rural men to be the only group where the majority was meeting recommended activity levels.

A major strength of our study is the detailed assessment of the various domains of daily activity. We were able to calculate total PAL, as well as examine energy expenditure in multiple activities of daily living. We found higher physical activity levels among men than women; our estimates of median energy expenditure (37.5 MET*h/d among men and 35.2 MET*h/d among women) were comparable to those reported from studies done in the U.S. and Germany [21,27]. A U.S. study of adults 18 years and older described mean energy expenditure of 36.8 MET*h/d among men, and 36.5 MET*h/d among women [21]; German participants, 13–80 years old had mean energy expenditure of 37.9 MET*h/d among men and 37.4 MET*h/d among women [27]. Differences in questionnaires and assumptions in calculating energy expenditure likely contribute to group differences; regardless, our findings do not support the long-held belief that activities of daily living are more physically demanding in developing countries.

The distinctions between rural and urban environments are becoming less coherent. In a recent analysis in China, Monda *et al*, used an urbanicity index, comprised of community and household level indicators, to demonstrate a linear association between light occupational activity and urbanization [9]. Similarly, in our population urban men were expending more energy in low intensity occupations than in moderate or vigorous occupations. However, we also found this trend towards light intensity occupations among nonagricultural-rural men living alongside the agricultural-rural men, indicating an increase in sedentary occupations in rural as well as urban areas.

Overall, activity levels among women were very low, but these results should be interpreted with caution. Among 19 rural women, a poor correlation (r = 0.04, ns) was observed between energy expenditure estimated by the questionnaire and by two non-consecutive days of 24-hour heart-rate monitoring [23]. This may be due to the low ability of heart-rate monitoring to quantify energy expenditure at lower intensity levels [28] or to limitations of the questionnaire. Eighty-one percent of women reported housewife as their primary occupation; this category likely represents a range of activities that we are not adequately capturing. Furthermore, among women in our cohort, the distinction between energy expended in occupation versus chores may not be appropriate, as the role of a housewife typically includes a variety of household and childcare chores. We found a greater amount of energy expended in chores among urban compared to rural women, likely due to more urban women working outside of the home, and thus being more likely to report additional chores. If some women were participating in higher intensity duties in the time reported as 'housewife,' both occupational energy expenditure and total energy expenditure would be underestimated. Nevertheless, we were able to describe reported time and energy expenditure in other domains of activity among women, notably the low energy expended in activities such as walking/cycling or in sports/exercise.

The obesity epidemic in developing countries is occurring consequential to large shifts in economic and cultural environments. Numerous factors can affect susceptibility to weight gain, including heritability, biological factors, and behaviors [1]. Globally, obesity appears to be more prevalent among women than men [1,29]. Parity is associated with overweight [30], and is likely contributing to the higher prevalence of overweight and obesity among women than men in our cohort, as 79% had 2 or more children, and 61% had three or more children. Nevertheless weight gain is fundamentally driven by energy intake exceeding expenditure. To correct the imbalance, energy intake can be decreased, energy expenditure increased, or some combination of both. The high prevalence of sedentariness is likely playing a significant role. This is further worrisome due to the range of health benefits of physical activity beyond simple energy balance. One must be cautious with efforts to reduce energy intakes in this population. Micronutrient deficiencies such as in iron, zinc and vitamin C are endemic and reduced food intakes will exacerbate these deficiencies unless accompanied by improvements in dietary quality.

Over the past several decades advancements in technology and infrastructure have contributed to decreasing participation in physically demanding agricultural and domestic activities [31], however, this Guatemalan population does not yet appear to be compensating by increasing participation in moderate or vigorous leisure-time activities, as only 36% of men and 5% of women reported participating in any sport or exercise. While there is little data from Latin America, a study from Brazil found a similar low prevalence of participation in sports or exercise, with only 18% of males and 8% of females reporting 30 minutes per day of leisure-time activity on at least one day per week [32]. A higher prevalence of participation was found among an urban Peruvian population: 45% at least once a week and 13% at least every other day among men, and 32% and 11% among women, respectively [13]. Analysis of data from the Third (U.S.) National Health and Nutrition Examination Survey, examining both leisure time and occupational physical activity data suggested that obesity was 50% lower among subjects who attained moderate physical activity for at least 30 min 5 or more d/wk, regardless of the level of occupational activity [33]. As sedentary occupations become more common, increasing participation in regular moderate intensity physical activities may help curtail the obesity epidemic unfolding in this population.

Despite only 36% of nonagricultural-rural and 24% of urban men meeting the PAL recommended for preventing obesity, 73% and 57%, respectively believed they were sufficiently active to promote health. This discrepancy is even more striking among women, where only 2% rural and 3% urban were meeting recommendations, yet 81% and 53%, respectively, believed they were sufficiently active. This is indicative of a dire need for public health campaigns in this population, outlining physical activity requirements for health promotion and obesity prevention, as well as suggestions on how to increase physical activity in daily life. Knowledge of physical activity patterns among populations is essential for the development of obesity prevention strategies.

## Conclusion

Among this population of Guatemalan adults, occupation was the largest contributor to total energy expenditure, primarily due to the amount of time spent in these activities. Overall, energy expenditure was low among the nonagricultural sector of the population. The findings in this paper indicate a need for increased energy expenditure among women and nonagricultural-rural and urban men; there is potential for significant gains in physical activity levels, particularly by promoting active leisure-time activities and decreasing time spent in sedentary activities.

## Competing interests

The author(s) declare they have no competing interests.

## Authors' contributions

ADS conceived of the study. COG carried out the coding and statistical analysis and drafted the manuscript. All authors participated in the study design, provided critical revision of the paper, and read and approved the final manuscript.
